# Dynamic PET Reveals Compartmentalized Brain and Lung Tissue Antibiotic Exposures

**DOI:** 10.21203/rs.3.rs-4096014/v1

**Published:** 2024-03-21

**Authors:** Sanjay Jain, Xueyi Chen, Bhavatharini Arun, Oscar Nino Meza, Mona Sarhan, Medha Singh, Byeonghoon Jeon, Kishor Mane, Maunank Shah, Elizabeth Tucker, Laurence Carroll, Joel Freundlich, Charles Peloquin, Vijay Ivaturi

**Affiliations:** Johns Hopkins University School of Medicine; Johns Hopkins University School of Medicine; University of Maryland School of Pharmacy; Johns Hopkins University School of Medicine; Johns Hopkins University School of Medicine; Johns Hopkins University School of Medicine; Johns Hopkins University School of Medicine; Rutgers New Jersey Medical School; Johns Hopkins University; Johns Hopkins University School of Medicine; Johns Hopkins University School of Medicine; Rutgers University; University of Florida College of Pharmacy; University of Maryland, Baltimore

## Abstract

Tuberculosis (TB) remains a leading cause of death, but antibiotic treatments for tuberculous meningitis, the deadliest form of TB, are based on those developed for pulmonary TB and not optimized for brain penetration. Here, we performed first-in-human dynamic ^18^F-pretomanid positron emission tomography (PET) studies in eight human subjects for three-dimensional, multi-compartmental *in situ* visualization of antibiotic concentration-time exposures (area under the curve – AUC), demonstrating preferential brain (AUC_tissue/plasma_ 2.25) versus lung (AUC_tissue/plasma_ 0.97) tissue partitioning. Preferential, antibiotic-specific partitioning into brain or lung tissues of antibiotics active against MDR strains were confirmed in experimentally-infected mice and rabbits, using dynamic PET with chemically identical antibiotic radioanalogs, and postmortem mass spectrometry measurements. PET-facilitated pharmacokinetic modeling predicted human dosing necessary to attain therapeutic brain exposures in human subjects. These data were used to design optimized, pretomanid-based regimens which were evaluated at human equipotent dosing in a mouse model of TB meningitis, demonstrating excellent bactericidal activity without an increase in intracerebral inflammation or brain injury. Importantly, several antibiotic regimens demonstrated discordant activities in brain and lung tissues in the same animal, correlating with the compartmentalized tissue exposures of the component antibiotics. These data provide a mechanistic basis for the compartmentalized activities of antibiotic regimens, with important implications for the development of antimicrobial regimens for meningitis and other infections in compartments with unique antibiotic penetration.

## Introduction

Achieving therapeutic antibiotic concentrations at infection sites is a prerequisite for effective treatments^1^. However, with few exceptions, current antibiotic dosing is based on plasma concentrations, without compartment-specific pharmacokinetic data at infection sites. Inappropriately low antibiotic tissue levels can select for antibiotic resistant bacteria, leading to treatment failure. Therefore, a growing number of studies and the U.S. Food and Drug Administration (FDA) support measuring antibiotic concentrations in infected tissues^2^. Importantly, antibiotic treatments for infections in compartments traditionally thought to have restricted antibiotic penetration, such as tuberculous meningitis (TB meningitis), the deadliest form of tuberculosis (TB)^3–5^, are not optimized, and continue to be based on those developed for pulmonary TB^3,4^, without compartment-specific pharmacokinetic data. There are substantial challenges in sampling deep tissue from live human subjects, especially from the brain, due to the associated risks and costs of these procedures. Moreover, sampling is generally limited to the most accessible lesion at a single time-point, precluding multi-compartment measures in the same subject or determination of concentration-time profiles^6^.

To overcome these limitations, we have developed novel, clinically-translatable tools for noninvasive, unbiased and *in situ* multi-compartmental, three-dimensional visualization of antibiotic concentration-time profiles^7,8^. Here, we performed first-in-human, whole-body, dynamic ^18^F-pretomanid positron emission tomography (PET) and computed tomography (CT) in eight human subjects (NCT05609552)^9^, to simultaneously assess brain and lung tissue antibiotic exposures as area under the concentration-time curve (AUC). ^18^F-Pretomanid is chemically identical to pretomanid, which is approved by the U.S. FDA for the treatment of multi-drug resistant (MDR) pulmonary TB in combination with bedaquiline and linezolid [BPaL - bedaquiline (B), pretomanid (Pa) and linezolid (L)]^10^. Further, a bidirectional process to integrate findings from human and animal studies was developed to optimize treatment regimens for TB meningitis (**Supplementary Fig. 1**). We performed whole-body dynamic PET with radioanalogs of antibiotics active against MDR strains (^18^F-pretomanid, ^18^F-sutezolid, ^18^F-linezolid and ^76^Br-bedaquiline) in experimentally-infected mice and rabbits to simultaneously assess brain and lung tissue AUCs. Direct measures of antibiotic levels in postmortem tissues from the animal studies were performed using mass spectrometry. PET-facilitated pharmacokinetic modeling and Monte Carlo simulations were used to predict tissue exposures and doses necessary to attain therapeutic brain exposures. These data were used to design optimized, pretomanid-based multidrug regimens which were tested in the mouse model of TB meningitis at human equipotent dosing. Bacterial burden in the brain and lung tissues were quantified as colony-forming units (CFU). Intracerebral inflammation was measured in live animals using ^124^I-DPA-713, a clinically translatable imaging biomarker of activated microglia and macrophages^11–14^, and complemented by postmortem analyses to assess neuroinflammation and markers of brain metabolism and injury.

## Results

### First-in-human ^18^F-pretomanid PET studies

**Figure 1:**
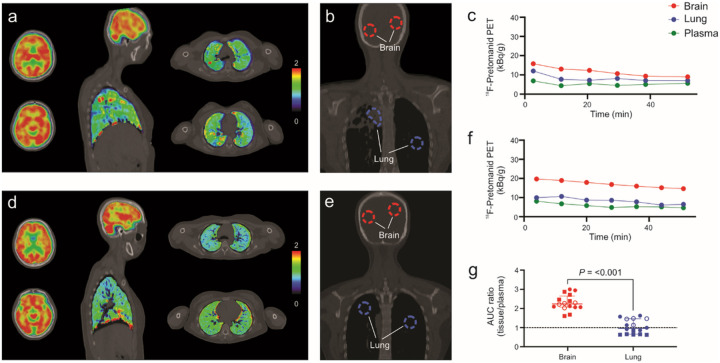
First-in-human dynamic ^18^F-pretomanid PET/CT studies in human subjects. Eight subjects were prospectively enrolled and imaged in accordance with the U.S. Food and Drug Administration guidelines (NCT05609552). **a**, ^18^F-Pretomanid PET area under the curve (AUC) heatmap overlay on computed tomography (CT) of a representative subject with pulmonary TB (subject 7). **b**, Coronal CT section from the same subject showing the volumes of interest (VOI) used to quantify the ^18^F-pretomanid PET signal and obtain time-activity curves from different tissues (panel **c**). **d**, ^18^F-Pretomanid AUC heatmap overlay on CT of representative healthy subject (subject 2). **e**, Coronal CT section from the same subject showing the volumes of interest (VOI) used to quantify the ^18^F-pretomanid PET signal and obtain time-activity curves from different tissues (panel **f**). **g**, ^18^F-Pretomanid (tissue-to-plasma) AUC_tissue/plasma_ ratios from all subjects demonstrate significantly higher brain versus lung tissue exposures. Circles and squares represent female and male subjects respectively. Empty shapes represent TB patients. n = 2 VOI per tissue per subject. Data are represented as median ± interquartile range. Statistical comparisons were made using a two-tailed Mann-Whitney U test.

Eight human subjects (six healthy volunteers^7^ and two newly diagnosed TB patients) (**Supplementary Table 1**) underwent, whole-body ^18^F-pretomanid PET/CT in accordance with the U.S. FDA guidelines for investigational drugs. Dynamic PET was performed for 50–60 min immediately after an intravenous injection of ^18^F-pretomanid (Fig. 1, **Supplementary Fig. 2**). Three-dimensional volumes of interest (VOI) were drawn in various compartments using the CT as a reference (Fig. 1b, e) to simultaneously measure time-concentration profiles in the brain and lung compartments (Fig. 1c, f). Tissue-to-plasma AUC ratios (AUC_tissue/plasma_) were calculated for each compartment as a measure of pretomanid exposures. Overall, 784 different measurements were made, of which 280 were from the brain and lung compartments and ^18^F-pretomanid distribution was consistent with its known metabolism. ^18^F-Pretomanid exposures were compartmentalized with median brain and lung AUC_tissue/plasma_ ratios of 2.25 [median; interquartile range (IQR), 2.08–2.54] and 0.97 (median; IQR, 0.67–1.47) respectively (*P* < 0.001) (Fig. 1g). ^18^F-pretomanid exposures were significantly lower in the cerebrospinal fluid (CSF) (ventricles) versus the brain parenchyma (**Supplementary Fig. 2i**; *P* = 0.027). There were no significant differences in brain or lung exposures between healthy or TB patients (*P* > 0.078).

### Animal studies with antibiotics active against MDR strains

**Figure 2:**
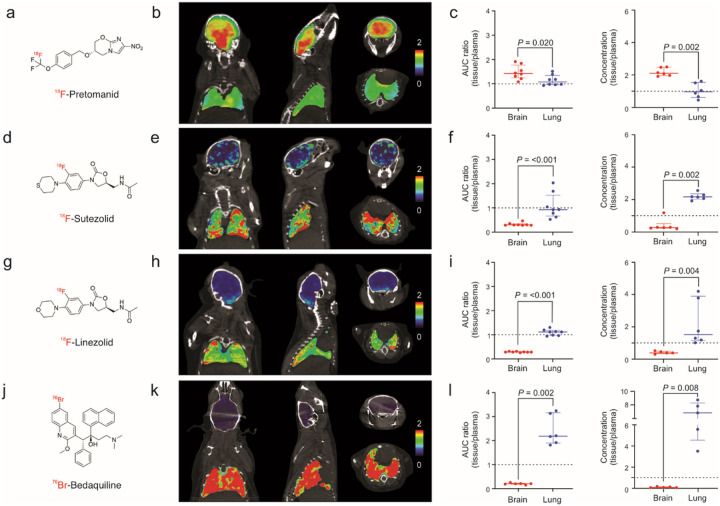
Compartmentalized antibiotic exposures in mouse studies. Radioanalog of four antibiotics active against multidrug-resistant (MDR) *Mycobacterium tuberculosis* strains were utilized in mouse studies. Each radioanalog is chemically identical to the respective parent antibiotic (panels **a**, **d**, **g**, **j**) and the radioisotope is retained within the major metabolite. Coronal, sagittal and transverse CT with PET AUC heatmap overlays (representing AUC_tissue/plasma_ ratio) from representative mice, demonstrating spatially compartmentalized antibiotic exposures in lung and brain compartments are shown for (**b**) ^18^F-pretomanid (AUC_0–60min_), (**e**) ^18^F-sutezolid (AUC_0–60min_), (**h**) ^18^F-linezolid (AUC_0–60min_), and (**k**) ^76^Br-bedaquiline (AUC_0–48h_). Tissue-to-plasma ratios obtained using dynamic PET in live animals (AUC ratio) and by mass spectrometry (MS) from postmortem (tissue-to-plasma ratio) brain (red) and lung (blue) tissues are respectively shown for (**c**) ^18^F-pretomanid PET [AUC_0–60min_ ratio, data from four animals; n = 8 brain volumes of interest (VOIs), n = 8 lung VOIs], pretomanid MS (tissue/plasma ratio, data from six animals; n = 6 brain, n = 6 lung samples), (**f**) ^18^F-sutezolid PET (AUC_0–60min_ ratio, data from four animals; n = 8 brain VOIs, n = 8 lung VOIs), sutezolid MS (data from six animals; n = 6 brain, n = 6 lung samples), (**i**) ^18^F-linezolid PET (AUC_0–60min_ ratio, data from four animals; n = 8 brain VOIs, n = 8 lung VOIs), linezolid MS (data from six animals; n = 5 brain, n = 6 lung samples), and (**l**) ^76^Br-Bedaquiline PET (AUC_0–48h_ ratio, data from three animals; n = 6 brain VOIs, n = 6 lung VOIs), bedaquiline MS (data from five animals; n = 5 brain, n = 5 lung samples). The horizontal dotted lines indicate tissue/plasma ratio of 1. Data are represented as median ± interquartile range. Statistical comparisons were made using a two-tailed Mann-Whitney U test.

Brain and lung exposures of radioanalogs of antibiotics active against MDR strains, ^18^F-pretomanid^7^, ^18^F-sutezolid, ^18^F-linezolid^15^, and ^76^Br-bedaquiline^16^ (all chemically identical to the parent antibiotic), were assessed using PET in experimentally-infected mice. ^18^F-Sutezolid was synthesized and validated as described in **Supplementary Fig. 3–5**. Dynamic PET was acquired for 60 min for ^18^F-pretomanid, ^18^F-sutezolid and, ^18^F-linezolid and 48 hours for ^76^Br-bedaquiline (due to its much longer half-life). PET plasma values were derived from blood (left ventricles). Coronal, sagittal and transverse brain and lung PET AUC_tissue/plasma_ heatmaps, quantification of PET data and the corresponding direct measures of antibiotic levels in postmortem tissues using mass spectrometry are shown (Fig. 2, **Supplementary Fig. 6**). All four antibiotics demonstrated compartmentalized exposures with significantly different brain and lung tissues exposures (*P* < 0.020). While ^18^F-pretomanid demonstrated median AUC_tissue/plasma_ ratio > 1 in both brain (1.41; IQR 1.27–1.60) and lung compartments (1.07; IQR 0.96–1.23), sutezolid, linezolid and bedaquiline had substantially lower median brain AUC_tissue/plasma_ ratios of 0.29 (IQR, 0.28–0.31), 0.28 (IQR, 0.28–0.29), and 0.21 (IQR, 0.19–0.21) respectively. In contrast, sutezolid, linezolid and bedaquiline had lung AUC_tissue/plasma_ ratios ~ 1, with bedaquiline demonstrating the highest AUC_tissue/plasma_ ratio of 2.19 (median; IQR, 1.98–2.90). Direct measures of antibiotic levels in postmortem tissues using mass spectrometry confirmed the antibiotic-specific, compartmentalized brain and lung exposures noted on PET imaging (*P* < 0.008). Mass spectrometry studies were also performed for moxifloxacin and pyrazinamide (**Supplementary Fig. 7**), which are also active against MDR strains. While pyrazinamide demonstrated excellent brain and lung penetration (median tissue/plasma ratio > 1), moxifloxacin demonstrated limited brain penetration (median tissue/plasma ratio 0.23; IQR 0.20–0.30) but excellent lung penetration (median tissue/plasma ratio 3.34; IQR 3.31–4.34). Similar experiments were performed in experimentally-infected rabbits confirming the limited brain exposures for ^18^F-sutezolid (median AUC_tissue/plasma_ ratio 0.36; IQR 0.27–0.44) and ^18^F-linezolid (median AUC_tissue/plasma_ ratio 0.23; IQR 0.19–0.25) (**Supplementary Fig. 8**). However, both antibiotics had median AUC_tissue/plasma_ ratio ~ 1 or greater in the lung compartment of rabbits. Direct measures of bedaquiline levels in postmortem tissues using mass spectrometry were also performed in rabbits (**Supplementary Fig. 9**), which confirmed the data from the mouse studies, demonstrating high lung, but much lower brain tissue and CSF levels.

### Pharmacokinetic modeling to predict human brain and lung tissues exposures

**Figure 3:**
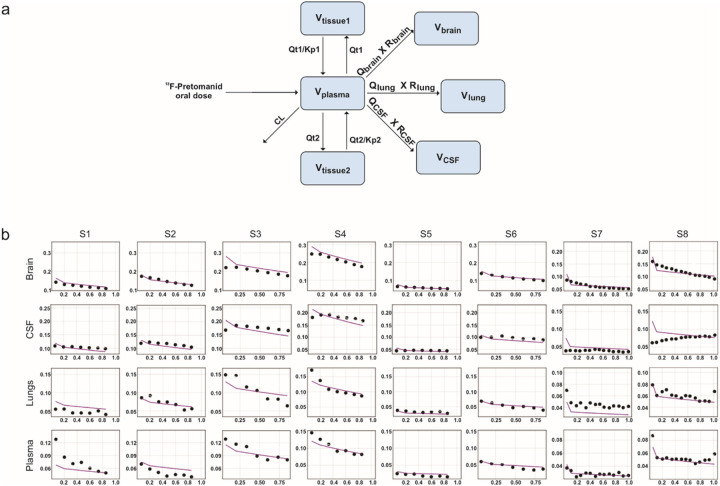
Pharmacokinetic model to predict human tissue exposures for pretomanid. **a**, Schematic representation of the model for pretomanid tissue penetration based on a physiology-based pharmacokinetic (mPBPK) framework. **b**, Observed (black dots) and individual model-predicted (purple line) for ^18^F-pretomanid exposures in different tissues [brain, CSF (measured in cerebral ventricles), lung and plasma (measured in left heart ventricle)] for the human subjects (S1-S8). Y-axis shows antibiotic exposure and x-axis shows time (hours). CL = plasma clearance, Kp = partition coefficient for the tissue compartment, Qt = blood flow rate to tissue, Qlung = blood flow rate to lungs, Qbrain = blood flow rate to brain, QCSF = blood flow rate to brain ventricles (CSF), Rbrain = penetration ratio for brain, RCSF = penetration ratio for CSF, Rlung = penetration ratio for lungs.

**Figure 4:**
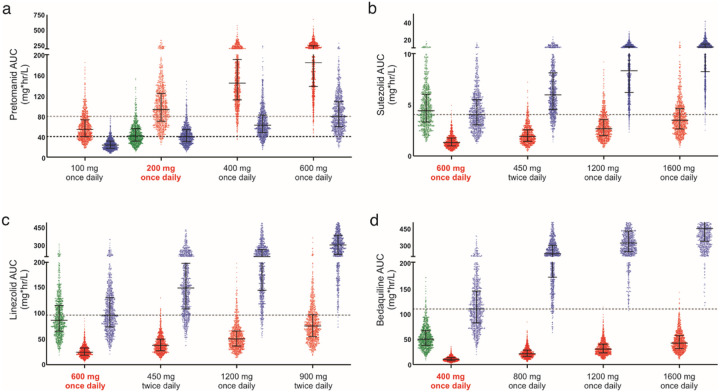
Monte Carlo simulations to predict humans tissue exposures. Monte Carlo simulations were performed in 1,000 virtual subjects for each antibiotic to predict tissue exposure area under the curve (AUC) in plasma (green), brain (red) and lung (blue) at various oral doses are shown for **a**, pretomanid, **b**, sutezolid, **c**, linezolid and, **d**, bedaquiline. The horizontal dotted black line represents target AUC for each antibiotic and represents the lung tissue exposures achieved with standard oral dosing (bolded red) in patients with pulmonary TB. A second dotted line is shown for pretomanid, representing a dose of 600 mg/day. Data are represented as median ± interquartile range.

We developed pharmacokinetic models for pretomanid, sutezolid, linezolid and bedaquiline that correctly predict human pharmacokinetic PET and published data (Fig. 3, **Supplementary Fig. 10**). Monte Carlo simulations (n = 1000 subjects for each antibiotic) were used to predict human brain and lung tissue exposures at various oral doses (Fig. 4). For pretomanid, the standard 200 mg/day oral dose achieved brain tissue exposures substantially higher than the lung tissue exposures. However, while the standard 600 mg/day dose for sutezolid and linezolid achieved therapeutic exposures in the lung tissues, it achieved subtherapeutic brain tissue exposures for both drugs. Several clinical trials are utilizing linezolid at 1,200 mg/day for TB meningitis^17,18^. However, while better than with the 600 mg/day dosing, this higher dose still achieves subtherapeutic brain tissue exposures for sutezolid or linezolid. Finally, bedaquiline brain tissue exposures were substantially lower than in the lung tissues at the standard 400 mg/day dose. Importantly, brain tissue exposures would remain subtherapeutic even at a dose of up to 1,600 mg/day.

### Optimized pretomanid-based multidrug regimens for TB meningitis

**Figure 5:**
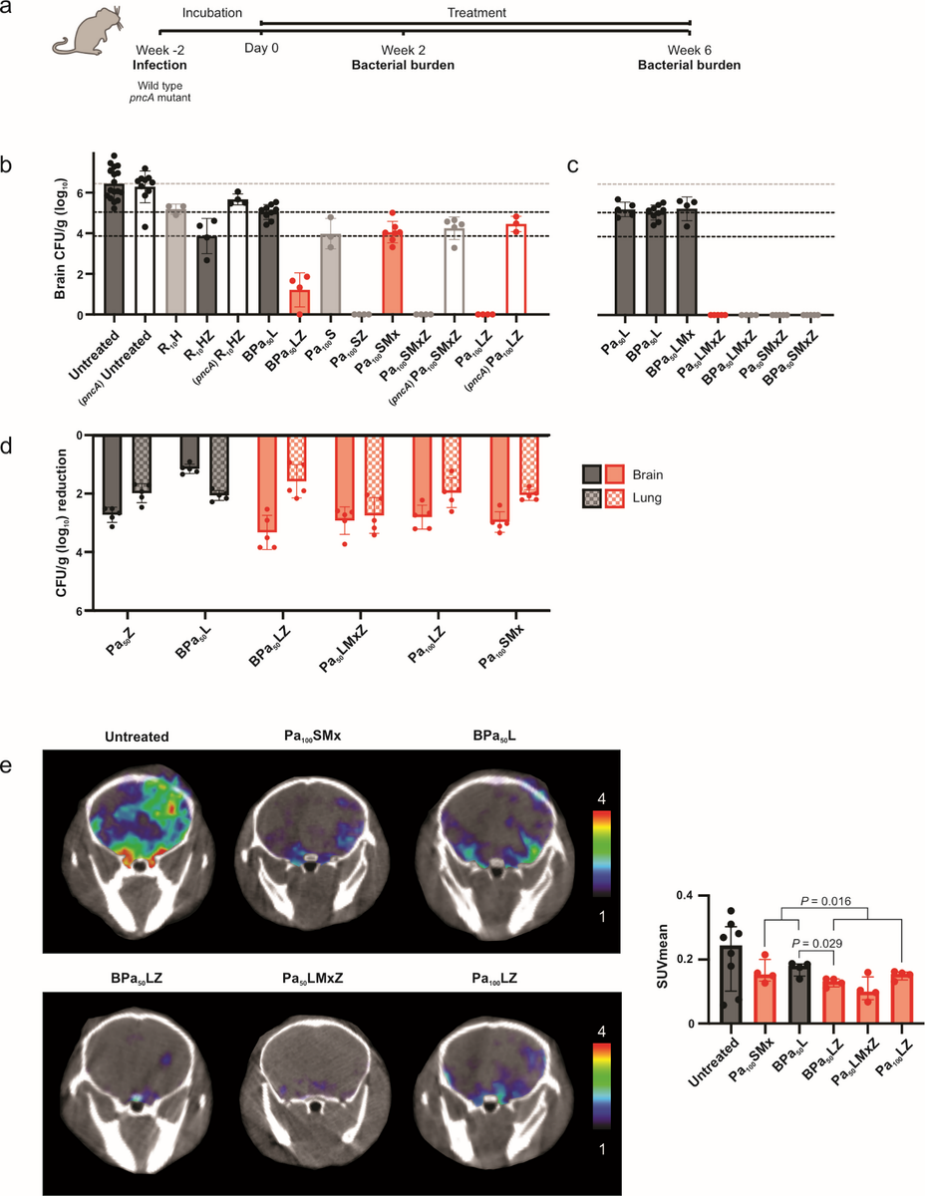
Optimized pretomanid-based multidrug regimens at human equipotent dosing. **a**, Two weeks after infection with *Mycobacterium tuberculosis,* mice with TB meningitis were randomly allocated to receive multidrug regimens via oral gavage at human equipotent dosing (R, rifampin; H, isoniazid; Z, pyrazinamide; B, bedaquiline; Pa_50_ or Pa_100_, pretomanid corresponding to a human dose of 200 mg/day or 400–600 mg/day respectively; L, linezolid; S, sutezolid; Mx, moxifloxacin). Selected pyrazinamide-containing regimens were tested in mice infected with pyrazinamide-resistant *M*. *tuberculosis (pncA* mutant). Red bars represent optimized regimens, grey bars represent other regimens and black bars represent reference regimens. All animals also received adjunctive dexamethasone, which is the standard of care for TB meningitis. **b**, Bacterial burden [colony-forming unit (CFU) per gram of brain tissue (log_10_) from whole brain] after six weeks of treatment (n = 3–14 mice/regimen). The bacterial burdens for reference regimens are shown as horizontal dotted lines for comparison; top grey is for untreated animals representing the starting bacterial burden, middle black is for the approved MDR regimen for pulmonary TB (BPa_50_L) and lower black is for the first-line, standard TB treatment for drug-susceptible TB meningitis (R_10_HZ). Regimens tested in mice infected with the *pncA* mutant are indicated by empty rectangles. **c**, Bacterial burden after six weeks comparing regimens with and without bedaquiline and/or pyrazinamide (n = 5–9 mice/regimen). **d**, Bacteria disseminate to the lung after the brain infection. Therefore, bactericidal activities of several antibiotic regimens in brain and lung tissues of the same animal were assessed. Data are shown as the reduction in whole brain (solid filled rectangle) and lung (checkered filled rectangle) organ CFU two weeks after treatment initiation (n = 4–5 mice/regimen). **e**, Mice underwent live imaging at two weeks after treatment initiation. Transverse ^124^I-DPA-713 PET/CT images of representative mice from different treatment regimens and untreated mice (left) and quantification of ^124^I-DPA-713 PET signal in the brain represented as standard uptake value mean (SUV_mean_) (n = 4–8 mice/group) (right) are shown. For CFU, data are represented as mean ± standard deviation and statistical comparisons were made using a two-tailed student t-test. For imaging, data are represented as median ± interquartile range. Statistical comparisons were made using a two-tailed Mann-Whitney U test.

Mice with experimentally-induced TB meningitis were randomly allocated to receive different multidrug regimens administered at human equipotent dosing via oral gavage (Fig. 5)^7,14^. All animals also received adjunctive dexamethasone, which is the standard of care for TB meningitis^19^. Bacterial burden was quantified in whole organs as CFU. Data at six weeks after initiation of multidrug regimens (Fig. 5b,c) show untreated animals representing the starting bacterial burden and two control regimens – first-line, standard TB treatment for drug-susceptible TB meningitis [standard-dose rifampin (human equipotent dose of 10 mg/kg/day, R_10_), isoniazid (H), and pyrazinamide (Z) – R_10_HZ] and the U.S. FDA approved treatment for MDR pulmonary TB [bedaquiline (B), pretomanid (Pa) and linezolid (L) – BPaL, here forth referred as BPa_50_L representing the human equipotent Pa dose used in mice equivalent to the standard human dose of 200 mg/day]. Pyrazinamide substantially improved the bactericidal activities of all MDR-TB regimens (*P* < 0.001), and this effect was abrogated in infections with pyrazinamide-resistant *Mycobacterium tuberculosis* (*pncA* mutant)^20^ (Fig. 5b). Addition of bedaquiline to the Pa_50_L regimen did not improve its activity (*P* = 0.460) (Fig. 5c). Several pretomanid-based multidrug regimens (red regimens in Fig. 5) were found to have bactericidal activities similar (or substantially better) than the first-line standard TB treatment (R_10_HZ) and substantially better than the BPa_50_L regimen. The additive effects of sutezolid (S) and linezolid were similar (*P* = 0.089) (Fig. 5, **Supplementary Fig. 11**). Since bacteria disseminate to the lung after a brain infection^14,21^, we were able to assess the activities of several antibiotic regimens in the brain and lungs simultaneously in the same animal (Fig. 5d). Data are shown as the reduction in whole organ CFU two weeks after initiation of treatment, with larger reductions indicating increased bacterial killing. Consistent with prior data, the BPa_50_L regimen has excellent activity in lung, but not in brain tissues. However, regimens optimized for TB meningitis (red regimens in Fig. 5) had better bactericidal activities in the brain compared to lung tissues. These data demonstrate discordant bactericidal activities in brain versus lung tissues in the same animal.

We assessed intracerebral inflammation in live animals using ^124^I-DPA-713 two weeks after initiation of treatments (Fig. 5e). While treatment with antibiotic regimens decreased intracerebral inflammation compared to untreated animals, pyrazinamide-containing regimens had significantly lower ^124^I-DPA-713 PET signal compared to the regimens without pyrazinamide (*P* = 0.016). Imaging studies in live animals were complemented by postmortem analyses to assess neuroinflammation and markers of brain metabolism and injury. Immunohistochemistry studies using Iba-1 + staining (measure of microglia) of brain tissues were consistent with the imaging findings with lower microglial density with antibiotic regimens (compared to untreated animals) and significantly lower microglial density in animals treated with pyrazinamide-containing regimens (*P* = 0.005; **Supplementary Fig. 12**). Brain and CSF cytokines (**Supplementary Fig. 13, Supplementary Fig. 14a**), CSF and plasma tryptophan levels (**Supplementary Fig. 14b, Supplementary Fig. 15**) and CSF and plasma levels of brain injury markers – glial fibrillary acidic protein (GFAP), neurofilament light chain (NEFL or Nfl), Tau and S100B (**Supplementary Fig. 14c, Supplementary Fig. 16**) demonstrated a similar trend.

## Discussion

TB remains a major threat to human health^22^. TB meningitis is a serious, life threatening form of TB, and current treatments prevent death or disability in less than half^5^. Importantly, the central nervous system (CNS) has more than one compartment [e.g. brain parenchyma and CSF], which are separated from the circulation by the blood-brain barrier (BBB) that limit penetration of many drugs. Despite knowledge that many antibiotics do not penetrate into the brain adequately and that immunopathology is the critical pathologic process, current TB meningitis treatment is not optimized and continues to be based on those developed for pulmonary TB^3,4^. Importantly, the alarming rise of MDR strains of *M. tuberculosis,* poses further challenges in the management of TB meningitis, as tissue pharmacokinetics and activities of newer antibiotics effective against MDR strains are not known. In fact, TB meningitis due to MDR strains is associated with the highest mortality^23,24^, with drug-resistance being an independent predictor of death^25^. In one report, mortality in TB meningitis patients with drug-resistance (67%) using current regimens was significantly higher than in those with drug-susceptible disease (24%, *P* < 0.001)^26^. Therefore, effective treatments against TB meningitis due to MDR strains are urgently needed.

Using whole-body, noninvasive and unbiased, dynamic PET imaging, we were able to obtain a rich dataset of concentration-time profiles in multiple compartments in three-dimensional space simultaneously, demonstrating compartmentalized brain and lung tissue exposures. Animal studies confirmed the compartmentalized pretomanid brain and lung tissue exposures noted in the human studies but also demonstrated antibiotic-specific compartmentalization, e.g. while pretomanid had higher brain versus lung tissue exposures, the opposite was noted for bedaquiline. Direct antibiotic measurements from postmortem animal tissues confirmed the findings from the PET studies. PET-facilitated pharmacokinetic modeling and Monte Carlo simulations were then used to predict antibiotic exposures in brain and lung tissues. Only pretomanid achieved therapeutic brain tissue exposures at the standard human oral dosing and bedaquiline brain tissue exposures remained subtherapeutic even at a dose four times the standard human oral dose. It should be noted that pretomanid up to 1,200 mg/day is tolerated well in humans^27^. Given its concentration-time dependent activity as well as animal studies demonstrating better bactericidal activities at human equivalent dose of 400–600 mg/day (versus 200 mg/day)^28^, higher pretomanid dosing could be considered^29^, especially when the exposures of other antibiotics in the regimen are predicted to be suboptimal. Pyrazinamide has excellent CSF^30,31^, and lung penetration and exposures correlate with treatment response in patients with MDR pulmonary TB with pyrazinamide-susceptible strains^32^. However, one study has reported increased mortality and neurological toxicity in TB meningitis with elevated CSF pyrazinamide^33^, but a mechanistic basis for this finding is not known and therefore the significance of this association is unclear. Nonetheless, pyrazinamide continues to be used worldwide as the standard first-line regimen for drug-susceptible TB meningitis for the first two months of treatments. Pyrazinamide has strong sterilizing activity critical for shortening pulmonary TB treatments and one study in children has demonstrated that regardless of disease stage at presentation, a 6-month pyrazinamide-containing regimen was more efficacious than 9 or 12 month regimens without pyrazinamide^34^. Although brain tissue exposures for pyrazinamide are unknown in humans, PET imaging in non-human primates with a chemically identical radioanalog of pyrazinamide demonstrated excellent brain tissue exposures^35^, which were consistent with our data (mass spectrometry) from the mouse studies. Similarly, while moxifloxacin (Mx), active against several MDR strains of *M. tuberculosis,* has excellent CSF penetration^36,37^, brain tissue exposures are not known and our data from mouse studies demonstrated limited brain (tissue/plasma ratio 0.23) but excellent lung tissue penetration (tissue/plasma ratio 3.34). Overall, these data suggested that pretomanid and pyrazinamide based multidrug regimens could be highly effective in TB meningitis. It should be noted that concomitant use of pretomanid and pyrazinamide led to hepatotoxicity-related treatment discontinuations in 6–7% of participants in one study for the treatment of pulmonary TB,^38^ with treatment-emergent elevations of alanine transaminase (ALT) greater than three times the normal limits in 10.8% of those treated with pretomanid-pyrazinamide regimens versus 8.6% and 5.6% in those treated with BPaL, or the first-line TB regimens. However, given the substantial benefits of pyrazinamide^32^, and the high mortality associated with TB meningitis, especially due to MDR strains, the risk-benefit comparison likely favors its use in TB meningitis. Finally, antibiotics such as sutezolid, linezolid and moxifloxacin that are active against MDR strains and have moderate brain tissue exposures, could provide additive activity when combined with other highly effective antibiotics.

Data from the Monte Carlo simulations were used to design optimized, pretomanid-based multidrug regimens in the mouse model of TB meningitis^7,14^, administered at human equipotent doses. While addition of pyrazinamide substantially improved the bactericidal activities of all MDR-TB regimens, as predicted, addition of bedaquiline did not. The additive effects of sutezolid and linezolid were similar, but sutezolid has a better safety profile at higher dosing (up to 1,600 mg/day)^39^, required to achieve better brain tissue exposures. Importantly, we developed several pretomanid-based multidrug regimens (BPa_50_LZ, Pa_100_LZ and Pa_50_LMxZ) active against TB meningitis due to MDR strains, with bactericidal activities substantially better than R_10_HZ or BPa_50_L regimens. However, given the high rates of pyrazinamide resistance amongst MDR strains^40^, we also developed a pyrazinamide-sparing regimen (Pa_100_SMx), which while not as bactericidal as pyrazinamide-containing regimens, was still as effective as the first-line standard TB treatment, and substantially better than the BPa_50_L regimen. Finally, we were able to assess the activities of several antibiotic regimens in the brain and lung compartments in the same animal, which demonstrated discordant bactericidal activities, corresponding to the compartmentalized tissue exposures of the component antibiotics in the regimen. While bactericidal activity is an important endpoint for pulmonary TB treatments, clinical outcomes in TB meningitis may also be closely associated with intracerebral inflammatory responses^41^. This was assessed using live imaging as well as postmortem analyses demonstrating that the optimized, pretomanid-based multidrug regimens did not increase intracerebral inflammation or markers of brain metabolism and injury. Specifically, pyrazinamide-containing regimens had significantly lower intracerebral inflammation. High CSF tryptophan levels are associated with increased mortality in patients with TB meningitis^42^, and CSF and plasma tryptophan levels were lower in mice treated with the optimized regimens. Similarly, elevated levels of brain injury markers [GFAP NEFL (or Nfl), Tau and S100B] in the CSF or plasma, are associated with poor outcomes in patients with brain damage^43^, and in TB meningitis^44^, and brain injury marker levels in CSF and plasma were lower in mice treated with the optimized regimens.

Our studies have some limitations. Healthy volunteers and patients with pulmonary TB were imaged with ^18^F-pretomanid PET, and pretomanid brain exposures were excellent in these subjects with presumably an intact BBB and healthy brain tissues. It is anticipated that pretomanid brain exposures would remain higher than those plasma in infected tissues^7^, or in the setting of a leaky BBB in patients with TB meningitis. Due to the high risks of work with MDR *M*. *tuberculosis* strains in the laboratory, antibiotic regimens active against MDR strains were evaluated in animals infected with the drug-susceptible *M. tuberculosis* H37Rv strain. This is the standard and accepted approach for TB drug development, even for antibiotic regimens active against MDR strains^45,46^. In fact, the vast majority of MDR-TB regimens currently being evaluated in clinical trials or in clinical use (BPaL) were originally developed in mouse models utilizing infections with the drug-susceptible *M*. *tuberculosis* H37Rv strain^10,20^. While ^18^F-pretomanid is chemically identical to the parent antibiotic, for PET studies it was administered at a microdose (ng-μg per subject) rather than at a therapeutic dose. However, several studies support that microdosing as a reliable predictor of the drug biodistribution at therapeutic doses^47,48^. Further, ^18^F-pretomanid and the other radiolabeled antibiotics were administered intravenously with the injection time corresponding to the plasma T_max_, and brain uptake reaching C_max_ (maximum concentration) within the first few minutes, enabling the first 60 min to adequately capture the pharmacokinetic profile. Of note, bedaquiline has a much longer half-life than the other antibiotics evaluated here, but given the much longer physical half-life (16 hours) of Br-76 utilized as the radiolabel for bedaquiline (by replacing the endogenous Br with Br-76), imaging could be performed for 48 hours after tracer injection^16^. Importantly, all radiolabels were introduced into the antibiotic to keep them chemically identical to the parent compound and retained even after their metabolism^7,15,16^.

In summary, we report first-in-human dynamic ^18^F-pretomanid PET/CT in eight human subjects to simultaneously measure brain and lung tissue exposures that demonstrated compartmentalized exposures. PET studies in live animal and direct antibiotic measurements in postmortem tissue samples validated these findings but also demonstrated preferential (AUC_tissue/plasma_ >1) antibiotic-specific partitioning into brain or lung tissues within the same subject, driven presumably by the physiochemical properties of each antibiotic. PET-facilitated pharmacokinetic modeling was used to design optimized, pretomanid-based multidrug regimens tested at human equipotent dosing in a mouse model of TB meningitis. We developed several multidrug regimens active against TB meningitis due to MDR strains, with bactericidal activities substantially better than R_10_HZ or BPa_50_L regimens, without an increase in intracerebral inflammation or markers of brain metabolism and injury. These optimized, pretomanid-based multidrug MDR regimens comprise antibiotics either already approved for human use or being assessed in clinical trials. Therefore, these regimens could be readily evaluated in clinical studies for TB meningitis. Importantly, several antibiotic regimens demonstrated discordant bactericidal activities in brain versus lung tissues in the same animal, correlating with compartmentalized tissue exposures of the component antibiotics visualized with PET and confirmed by postmortem mass spectrometry. Our data provide a mechanistic basis for the discordant activities of antibiotic regimens for pulmonary TB and TB meningitis. Our approach is highly generalizable and has major implications for antimicrobial drug development and compartment-specific optimization of regimens, especially for meningitis and other infections in compartments with unique antibiotic penetration.

## ONLINE METHODS

All protocols were approved by the Johns Hopkins University Biosafety, Radiation Safety, Animal Care and Use (MO19M382, RB22M351, RB19M24) and Institutional Review Board (IRB00303845) Committees. The clinical study was registered on clinicaltrials.gov (NCT05609552)^9^.

### Human studies

^18^F-Pretomanid was synthesized as a sterile solution with high specific activity (45.54 ± 14.86 GBq/μmol) and high radiochemical purity by the Johns Hopkins PET Center. These studies were performed in accordance with the U.S. FDA Radioactive Drug Research Committee guidelines^49^. Eight human subjects (six healthy volunteers^7^ and two newly diagnosed TB patients) were prospectively recruited from the Johns Hopkins Hospitals between May 2022 to December 2023 using the following inclusion criteria (**Supplementary Table 2**). Written informed consent was obtained from each subject, physical examination performed by a trained physician and screening laboratory tests performed and reviewed by the study principal investigator to confirm eligibility. On the day of imaging, a low-dose CT, selected to minimize the radiation exposure, was performed. An intravenous injection of ^18^F-pretomanid (358.90 ± 12.64 MBq) was administered, followed immediately by a dynamic PET/CT (Biograph mCT, Siemens, Washington, DC) for 40–60 min utilizing a multi-bed protocol (mid-abdomen to the skull vertex), operating in three-dimensional emission acquisition mode and using CT for attenuation correction. Adverse events were assessed immediately after imaging and 20–25 days after the imaging study via a telephone interview. Blood data was obtained by placing a VOI in the left ventricle of the heart and corrected to plasma using hematocrit and pretomanid red blood cell (RBC) partition coefficients.

### Animal studies

Female C3HeB/FeJ mice (6–8 weeks old, Jackson laboratory) or male and female New Zealand White rabbits (5–7 day old, Robinson Services Inc.) were intraventricularly infected using titrated frozen stocks of *M. tuberculosis* H37Rv or mutants as described previously^14^. Another set of male and female New Zealand White rabbits weighing 2.5–3.5 kg (Charles River, Wilmington, MA) were aerosol infected with *M. tuberculosis* H37Rv and noninvasively monitored by CT (CereTom, Neurologica, Danvers, MA) for the development of pulmonary TB lesions as described previously^8^.

#### Imaging

The antibiotic radiotracers were synthesized as described previously^7,15,16^. Details ^18^F-sutezolid synthesis, including the precursor and intermediates, and *in vivo* characterization, are described in Supplementary Materials. ^124^I-DPA-713 was purchased from 3DImaging, LLC through a research contract^50^. Animal studies were performed within 10 days of starting multidrug regimens. The radiotracers were administered intravenously to *M*. *tuberculosis*-infected animals at doses outlined in **Supplementary Table 3**. Animals were imaged inside sealed biocontainment containers [compliant with biosafety level (BSL)-3 containment]^7,51,52^. PET/CT acquisition were performed using the nanoScan PET/CT (Mediso, Arlington, VA). Blood data was obtained by placing a VOI in the left ventricle of the heart and corrected to plasma using hematocrit and RBC partitioning for each antibiotic^7,21^ (and **Supplementary Tables 4, 5**). For ^76^Br-bedaquiline mouse studies, raw imaging data from Ordonez *et al*^16^ were reanalyzed and corrected to plasma using hematocrit and bedaquiline RBC partition coefficients (Supplemental Table 5). For ^18^F-linezolid rabbit studies, raw imaging data from Tucker *et al*^21^ were reanalyzed to include lung tissue exposures.

#### Antimicrobial treatments

Drug stocks were prepared and administered five days a week via oral gavage at human equipotent dosing (**Supplementary Table 6**)^7^. Dexamethasone was administered intraperitoneally. Bacterial burden was quantified in whole organs as CFU at two and six weeks after initiation of treatment using 7H11 plates supplemented with activated charcoal. For aerosol infected rabbits, the BPaL regimen was administered five days a week via oral gavage at human equipotent dosing for two weeks.

#### Mass spectrometry

Tissues were collected at plasma T_max_ for each antibiotic in animals that had received at least 10 days of multidrug regimens. Antibiotics and their metabolites were quantified using validated ultra-high-performance liquid chromatography (UPLC) and tandem mass spectrometry (LC–MS/MS) at the Infectious Diseases Pharmacokinetics Laboratory of the University of Florida. The lower limits of detection were 0.05, 0.10, 0.10, 0.30, 0.03, 0.12, 0.50 and 0.20 μg/mL for bedaquiline, M2, pretomanid, linezolid, sutezolid, PNU-101603, pyrazinamide and moxifloxacin, respectively

#### Cytokines, tryptophan, and brain injury markers

Samples were collected and stored at −80°C until analysis. Cytokines were analyzed using the Luminex Multiplex assays by the Johns Hopkins University Oncology Human Immunology Core. Brain injury markers [GFAP (ab233621), NEFL (ab288182), cleaved Tau (ab269557), and S100B (OKEH00537)] and cellular metabolite tryptophan (KA1916) were quantified using their respective ELISA kits (Abcam, Novus Biologicals and Aviva systems biology).

### Image analysis

Human images were analyzed using Mirada XD^™^ 3.6.8 (Mirada Medical) and PMOD version 3.402 (PMOD Technologies LLC) while the animal images were analyzed using VivoQuant 2020 (Invicro). Three-dimensional volumes of interest (VOIs) were drawn using the CT as a reference and the PET data extracted as time-activity curves (TACs), which were used to calculate tissue AUCs and represented as AUC_tissue/plasma_ ratios^7,8^. PET activity was converted from tissue volume to tissue mass using tissue density from the Hounsfield units (CT). Heatmap overlays were created using AMIRA 5.2.1 (Visage Imaging, Inc.) and AMIDE 1.0.6 (Andreas Loening).

### Pharmacokinetic modeling

The pharmacokinetic modeling of bedaquiline, pretomanid, linezolid, and sutezolid was based on a published minimal physiology-based pharmacokinetic (mPBPK) framework, which included plasma and lung compartments^53^. This model was extended to include the CNS by integrating human physiological parameters^54^. Antibiotic-specific parameters (**Supplementary Tables 7–10**) were sourced from existing literature models^53,55^. For sutezolid, these parameters were fine-tuned utilizing digitized pharmacokinetic data. Drug uptake in the lungs and brain was explained using the effect compartment model, where exposure within these compartments is defined by the drug transport rates and penetration ratios. The transport rates are governed by blood flow rates, which in turn depend on the volume of the respective organ. Antibiotic penetration coefficients were calculated from the PET data as tissue-to-plasma AUC ratios (AUC_tissue/plasma_). Since the penetration coefficients are derived from drug concentration in the target tissue compartments, this inherently accounts only for the free drug that is not bound to plasma proteins and is free to enter the tissue compartments. Any possible drug accumulation over the duration of treatment was investigated for all antibiotics. The models were validated through their ability to accurately predict the observed human ^18^F-pretomanid PET data or the published (digitized) human pharmacokinetic data (**Supplementary Table 11**).

Monte Carlo simulations were performed based on the mPBPK to predict tissue exposure at various oral doses for the antibiotics under study. One thousand virtual subjects were simulated for each antibiotic, at several dose levels to cover a comprehensive range. The simulations integrated a 40% interindividual variability, anticipating significant differences in drug exposures among patients in clinical settings. Classically therapeutic targets for antibiotics are chosen based on free drug levels achieved in the tissue of interest. However, data on free drug levels in brain tissue is lacking for most antibiotics and it is also difficult to estimate free drug tissue levels for highly protein bound antibiotics (e.g. pretomanid and bedaquiline). Fortunately, there is excellent data correlating antibiotic exposures required for optimal bacterial killing in lung tissues^28,53,56–59^, and we therefore chose lung tissue antibiotic exposures achieved with standard oral dosing for patients with pulmonary TB, as the therapeutic target for optimal brain exposures. All analyses were performed using Pumas^®^ version 2.0 (Pumas-AI).

### Statistical Analysis

Data were analyzed using Prism 10.1.1 (GraphPad). The linear trapezoidal rule was used to calculate PET-derived AUCs. Bacterial burden (CFU) are represented on a logarithmic scale (base 10) as mean ± SD and comparisons were made using a two-tailed student t-test. All other data are represented as median ± IQR and comparisons were made using a two-tailed Mann-Whitney U test. *P* values ≤ 0.05 were considered statistically significant.

## Data Availability

All data are available in the main text or the supplementary materials. Source data are provided with this paper.
